# Perceived neighborhood environment and multidimensional pain burden among U.S. adults

**DOI:** 10.3389/fpubh.2026.1844301

**Published:** 2026-07-08

**Authors:** Zhixiao Xu, Siyuan Chen, Chengshui Chen, Jiaxing Ruan

**Affiliations:** 1 Department of Pulmonary and Critical Care Medicine, The First Affiliated Hospital of Wenzhou Medical University, Wenzhou, China; 2 Musculoskeletal Rehabilitation Department, Taizhou Rehabilitation Hospital, Taizhou, China; 3 Key Laboratory of Interventional Pulmonology of Zhejiang Province, Wenzhou, China; 4 Zhejiang Province Engineering Research Center for Endoscope Instruments and Technology Development, The Quzhou Affiliated Hospital of Wenzhou Medical University, Quzhou People’s Hospital, Quzhou, China; 5 Department of Pulmonary and Critical Care Medicine, Taizhou Central Hospital, Taizhou University Hospital, Taizhou, China

**Keywords:** built environment, chronic pain, neighborhood walkability, pain severity, pain-related limitations

## Abstract

**Background:**

Chronic pain constitutes a multidimensional public health crisis. Environmental determinants may significantly influence pain experiences, yet the specific impact of perceived neighborhood walkability, infrastructure, and safety remains underexplored.

**Methods:**

We analyzed data from 31,568 U.S. adults in the 2020 National Health Interview Survey (NHIS), utilizing Multiple Imputation by Chained Equations (MICE) to address missingness. To minimize conceptual overlap and Type I error, pain frequency was designated as the primary outcome, with pain intensity and functional interference as secondary outcomes. Exposures were operationalized as a composite Total Neighborhood Score (higher = more favorable) and three functional domains (Safety, Access, and Leisure). We employed survey-weighted proportional odds ordinal logistic regression to estimate adjusted odds ratios (aORs) and 95% confidence intervals (CIs). A Joint Domains Model was constructed to simultaneously analyze the three domains, with comprehensive covariate adjustment informed by a Directed Acyclic Graph.

**Results:**

In fully adjusted models, a higher Total Neighborhood Score was significantly associated with lower cumulative odds of experiencing more frequent pain (aOR = 0.95, 95% CI: 0.94–0.97). When evaluated in the Joint Domains Model, safety domain demonstrated the strongest independent protective association against frequent pain (aOR = 0.84, 95% CI: 0.80–0.89). Leisure domain also independently correlated with reduced pain frequency (aOR = 0.95, 95% CI: 0.91–1.00), whereas utilitarian access domain was attenuated after mutual adjustment. Consistent independent protective effects of safety and leisure were observed across all secondary outcomes.

**Conclusion:**

Perceived neighborhood safety and access to restorative leisure environments are independently associated with a reduced multidimensional burden of pain among U.S. adults. Integrating interdisciplinary urban planning strategies, such as traffic calming, crime reduction, and accessible green spaces, into public health initiatives may serve as essential non-pharmacologic interventions to mitigate chronic pain.

## 1 Introduction

Chronic pain poses a noticeable public health impact and constitutes a multidimensional health crisis, affecting over 20% of adults globally and ranking as the foremost contributor to years lived with disability (1). Disparities in pain experience and management have been observed across sociodemographic groups, including by age, gender (2), race/ethnicity (3), and income level (4). To fully understand these disparities, contemporary pain research has increasingly adopted the biopsychosocial perspective, which posits that the experience of pain is not merely a biological phenomenon (nociception), but is profoundly modulated by psychological states, social contexts, and environmental exposures (5).

Within this biopsychosocial framework, emerging evidence suggests that the built and social environments of neighborhoods act as critical upstream determinants of health (6–8). While early research predominantly focused on neighborhood walkability in relation to cardiometabolic outcomes, a growing, yet nascent, body of literature links adverse neighborhood environments to increased pain frequency and severity (9). However, the mechanisms underlying this association extend far beyond the conventionally cited pathway of physical activity. While walkable environments facilitate exercise, a recommended non-pharmacologic approach for managing musculoskeletal pain (10), neighborhoods also harbor complex environmental and psychosocial exposures. Chronic exposure to neighborhood hazards, such as crime, heavy traffic, and physical disorder, triggers chronic psychological stress. This sustained stress can lead to allostatic load, neuroendocrine dysregulation, and central sensitization, thereby amplifying pain perception and chronicity (11). Conversely, neighborhoods equipped with restorative spaces (e.g., quiet paths, green spaces) may buffer psychological stress and promote neurobiological recovery (12).

Despite the growing theoretical interest in spatial epidemiology and pain, substantial gaps remain. First, many existing studies rely heavily on objective spatial data (e.g., GIS-derived walkability scores) rather than subjective neighborhood appraisals. Yet, evidence suggests that individuals’ perceived neighborhood characteristics often exert a more direct influence on health behaviors and physiological stress responses than objective metrics (13). The cognitive appraisal of one’s environment, such as the fear of traffic or crime, can induce hypervigilance, which directly exacerbates the subjective experience of pain. Second, most existing research has focused on localized or urban-specific samples, limiting generalizability (14). Third, previous studies frequently evaluate environmental features in isolation, ignoring the conceptual overlap between infrastructure, access to amenities, and safety, which obscures the independent drivers of pain.

To address these gaps, we utilized nationally representative data from the 2020 cycle of the National Health Interview Survey (NHIS). Using a highly streamlined analytic strategy, we aimed to evaluate the association between the perceived neighborhood environment and multidimensional pain outcomes (frequency, severity, and interference) among U.S. adults. To isolate independent environmental drivers and reduce confounding, we evaluated a composite neighborhood environment score and subsequently grouped specific features into three distinct functional domains: Safety, Access, and Leisure. We hypothesized that within the biopsychosocial model, protective neighborhood domains, particularly perceived safety and restorative leisure spaces, would be independently associated with a lower burden of pain, even after rigorously adjusting for sociodemographic, psychological, and clinical covariates. Understanding these relationships may inform urban planning and public health strategies aimed at reducing pain and improving population wellbeing.

## 2 Materials and methods

### 2.1 Data source

This study utilized data from the 2020 cycle of the NHIS, a nationally representative survey administered annually by the National Center for Health Statistics (NCHS). The 2020 NHIS adult sample consisted of two components: a cross-sectional group of 21,153 newly sampled adults, and a longitudinal follow-up group of 10,415 respondents who had participated in the 2019 survey. Combined, the adult dataset comprised 31,568 individuals.

The proportion of missing data for all variables included in the final multivariable models is detailed in Supplementary Table S1. To confirm the validity of the multiple imputation procedure, we compared the distributions of core variables and covariates with the highest missingness rates before and after imputation. The distributions remained highly consistent, indicating that the Multiple Imputation by Chained Equations (MICE) algorithm effectively preserved the underlying data structure without introducing bias (Supplementary Table S2).

Data were derived from the 2020 NHIS, which was conducted during the COVID-19 pandemic. Because in-person interviews were largely suspended and data collection relied heavily on telephone interviewing, response rates were lower than in previous survey years. The NHIS sampling weights were adjusted by NCHS to account for nonresponse and maintain national representativeness.

All NHIS protocols were approved by the NCHS Research Ethics Review Board. Prior to participation, households received advance notification by mail, and verbal informed consent was obtained during the interview process. Detailed survey methodology and publicly available data files can be accessed via the NHIS website: https://www.cdc.gov/nchs/nhis.htm.

### 2.2 Measures

#### 2.2.1 Neighborhood environmental measures

Neighborhood environment perceptions were assessed using a series of questions included in the 2020 NHIS, sponsored by the National Cancer Institute and the Centers for Disease Control and Prevention. Respondents answered eight questions designed to capture walkability and safety characteristics of their local environments. These included: (1) the availability of roads, sidewalks, paths, or trails for walking; (2) whether most streets in the neighborhood have sidewalks; (3) access to places conducive to relaxation and stress relief; (4) proximity to shops, stores, or markets; (5) access to bus or transit stops; (6) access to entertainment or community services (e.g., libraries, movie theaters, places of worship); (7) whether traffic makes walking unsafe; and (8) whether crime makes walking unsafe.

The first two items were categorized as indicators of pedestrian infrastructure, items 3 through 6 reflected access to various land uses, and the final two items assessed perceived safety barriers to walking. All variables were binary (yes/no) and were analyzed individually in regression models to examine their associations with pain-related outcomes. The Total Neighborhood Score (higher = more favorable) represents the composite sum of the 8 binary items, while secondary exposure scores represent specific functional domains (Table 1). All 8 items were coded or reverse-coded such that a value of ‘1’ consistently indicated a positive or safe neighborhood characteristic. These items were then summed to calculate a Total Neighborhood Score ranging from 0 to 8, where higher scores represent a cumulative, more favorable, supportive, and safer perceived neighborhood environment.

**Table 1:** Operational definitions and scoring strategy for perceived neighborhood environment exposures (NHIS 2020).

Exposure variable	Item description	Binary recoding strategy
Primary exposure		
Total Neighborhood Score (range: 0–8)	*Sum of binary items (1 point for each favorable condition)* (Higher = More Favorable)	
Roads/streets suitable for walking (s1)	Roads/streets in neighborhood suitable for walking	Yes = 1, No = 0
Sidewalks present (s2)	Sidewalks present in neighborhood	Yes = 1, No = 0
Walk for relaxation (s3)	Walk for relaxation	Yes = 1, No = 0
Shops nearby (s4)	Shops within walking distance	Yes = 1, No = 0
Transit stops nearby (s5)	Transit stops nearby	Yes = 1, No = 0
Walk for fun/leisure (s6)	Walk for fun or leisure	Yes = 1, No = 0
Traffic safety (s7)	Neighborhood unsafe from traffic	No = 1, Yes = 0
Crime safety (s8)	Neighborhood unsafe from crime	No = 1, Yes = 0
Secondary exposures		
1. Safety score (range: 0–2)	*Sum of traffic (s7) and crime (s8)* (Higher = Safer)	—
2. Access score (range: 0–3)	*Sum of sidewalks (s2), shops (s4), transit (s5)* (Higher = Better Access)	—
3. Leisure score (range: 0–2)	*Sum of relaxation (s3) and fun (s6)* (Higher = More Restorative)	—

NHIS, National Health Interview Survey. All exposure variables were derived from self-reported responses to the adult survey module. The Total Neighborhood Score (higher = more favorable) represents the composite sum of the 8 binary items, while secondary exposure scores represent specific functional domains.

#### 2.2.2 Outcome assessment: pain-related measures

Self-reported pain was evaluated using a series of questions regarding participants’ experiences over the past 3 months. Participants were asked how often their pain limited their life or work activities, with response options of “never,” “some days,” “most days,” or “every day.” They were also asked about the intensity of pain during their most recent experience, categorized as “a little,” “somewhere in between,” or “a lot.” In addition, participants were asked how often their pain impacted their family members or significant others, with the same frequency response options. These measures provided insight into both the frequency and severity of pain and its broader effects on social and family life.

#### 2.2.3 Covariates

Covariate selection was strictly guided *a priori* by a Directed Acyclic Graph (DAG) using the dagitty package to identify the minimal sufficient adjustment sets (age, socioeconomic status, urban/rural residence). To further improve precision, objective clinical factors (history of arthritis, cancer, mental health diagnoses, and a cardiovascular risk score) were included. Notably, ‘self-reported general health status’ was deliberately excluded from the final multivariable models. Diagnostic evaluations indicated that global health status conceptually overlaps with and acts as a subjective proxy for specific chronic conditions (e.g., arthritis, depression), and its inclusion would introduce severe over-adjustment and bias the coefficients (Supplementary Figure S1).

### 2.3 Statistical analysis

Missing data were rigorously addressed using MICE to generate *m* = 20 imputed datasets. To ensure distributional accuracy, imputation algorithms were specifically matched to variable types: predictive mean matching (pmm) for continuous variables, logistic regression (logreg) for binary variables, polytomous logistic regression (polyreg) for nominal variables, and proportional odds models (polr) for ordinal variables. Complex survey design variables were forced into the predictor matrix to ensure the imputed values reflected the underlying sampling design.

Recognizing the ordinal nature of our pain outcomes, we utilized survey-weighted Proportional Odds Ordinal Logistic Regression models, generating cumulative Odds Ratios (ORs) and 95% Confidence Intervals (CIs). Unadjusted models include only the respective exposure variable and adjusted models that adjusted by age, sex, education, poverty level, region, urban/rural residence, marital status, arthritis, cancer, mental health diagnoses, diabetes, hypertension, smoking status, obesity, and physical activity were performed. To limit the risk of Type I error associated with multiple comparisons, we emphasize 95% CIs and have avoided reporting a proliferation of *p*-values. We explicitly designated pain frequency as the primary outcome, and the remaining metrics (pain intensity, limitations on work/life, and family impact) as secondary outcomes.

Joint Domains Model simultaneously incorporated all three environmental domains (Safety, Access, Leisure) into a single multivariable equation. This joint approach rigorously mutually adjusts for the domains, isolating their independent protective effects against pain. All models were adjusted for the minimal sufficient adjustment set identified via our DAG.

Furthermore, the proportional odds assumption was validated via a sensitivity analysis using Advanced Partial Proportional Odds Models (PPOM).

All analyses were conducted using R software (version 4.5.3).

## 3 Results

### 3.1 Characteristics of the study population

A total of 31,568 adults from the 2020 NHIS were included in the study. The proportion of missing data across the analytical variables ranged from 0.0 to 5.5% (Supplementary Table S1). Missing values were successfully addressed using multiple imputation, which robustly preserved the original distributions of the sociodemographic and health-related characteristics (Supplementary Table S2). In the overall imputed sample, 36.3% of participants reported never experiencing pain, while 39.9, 8.9, and 15.0% reported experiencing pain on some days, most days, and every day, respectively.

As detailed in Table 2, the majority of the study population were aged 18–44 years (46%), female (52%), married or partnered (59%), and residing in urban areas (86%). Distinct sociodemographic and clinical trends were observed across the pain frequency categories. Participants reporting daily pain (“Every day”) were more likely to be older, female, and have lower educational attainment compared to those who never experienced pain. Furthermore, a substantially higher burden of chronic conditions was observed among individuals with daily pain. Most notably, the prevalence of arthritis (59% vs. 5.4%), mental health disorders (40% vs. 13%), and obesity (75% vs. 61%) were markedly elevated in the daily pain group compared to the “Never” pain frequency group.

**Table 2:** Estimates of sociodemographic and clinical characteristics of the study population by pain frequency, United States, 2020^a^.

Characteristic	Frequency of pain				Overall
	Never	Some days	Most days	Every day	
	*N* = 11,275	*N* = 12,429	*N* = 2,761	*N* = 4,661	*N* = 31,126
Age, years					
18–44	5,200 (57%)	4,150 (44%)	742 (38%)	640 (20%)	10,732 (46%)
45–64	3,395 (28%)	4,159 (33%)	1,011 (37%)	1,884 (43%)	10,449 (32%)
≥65	2,680 (15%)	4,120 (23%)	1,008 (26%)	2,137 (36%)	9,945 (22%)
Missing	0 (0%)	0 (0%)	0 (0%)	0 (0%)	0 (0%)
Sex/gender					
Men	5,569 (51%)	5,574 (47%)	1,155 (45%)	2,028 (46%)	14,326 (48%)
Women	5,706 (49%)	6,855 (53%)	1,604 (55%)	2,633 (54%)	16,798 (52%)
Missing	0 (0%)	0 (0%)	2 (0.2%)	0 (0%)	2 (<0.1%)
Educational attainment^b^					
<High school	986 (13%)	1,067 (12%)	272 (15%)	739 (21%)	3,064 (14%)
Bachelor’s degree or higher	4,959 (32%)	5,172 (32%)	946 (25%)	1,156 (17%)	12,233 (29%)
High school graduate	2,323 (25%)	2,559 (25%)	641 (26%)	1,181 (28%)	6,704 (26%)
Some college	2,950 (29%)	3,585 (30%)	893 (33%)	1,556 (33%)	8,984 (30%)
Missing	57 (0.7%)	46 (0.6%)	9 (0.7%)	29 (0.9%)	141 (0.7%)
Family income as a percentage of FPL^c^					
<100% FPL	852 (9.1%)	863 (8.1%)	322 (12%)	724 (16%)	2,761 (9.9%)
100–199% FPL	8,744 (73%)	9,682 (75%)	1,960 (69%)	2,850 (61%)	23,236 (72%)
≥200% FPL	1,679 (18%)	1,884 (16%)	479 (19%)	1,087 (23%)	5,129 (18%)
Missing	0 (0%)	0 (0%)	0 (0%)	0 (0%)	0 (0%)
Urban–rural classification					
Rural	1,379 (12%)	1,840 (14%)	516 (16%)	958 (20%)	4,693 (14%)
Urban	9,896 (88%)	10,589 (86%)	2,245 (84%)	3,703 (80%)	26,433 (86%)
Missing	0 (0%)	0 (0%)	0 (0%)	0 (0%)	0 (0%)
Marital status					
Married/partnered	6,110 (58%)	6,763 (61%)	1,469 (61%)	2,190 (59%)	16,532 (59%)
Single/other	5,165 (42%)	5,666 (39%)	1,292 (39%)	2,471 (41%)	14,594 (41%)
Missing	0 (0%)	0 (0%)	0 (0%)	0 (0%)	0 (0%)
Arthritis^d^					
No	10,390 (95%)	9,322 (80%)	1,435 (60%)	1,703 (41%)	22,850 (79%)
Yes	876 (5.4%)	3,098 (20%)	1,320 (39%)	2,947 (59%)	8,241 (21%)
Missing	9 (<0.1%)	9 (<0.1%)	6 (0.2%)	11 (0.2%)	35 (0.1%)
Cancer^d^					
No	10,298 (94%)	10,731 (90%)	2,290 (86%)	3,705 (83%)	27,024 (90%)
Yes	966 (5.6%)	1,691 (10%)	470 (13%)	948 (17%)	4,075 (9.6%)
Missing	11 (<0.1%)	7 (<0.1%)	1 (<0.1%)	8 (0.1%)	27 (<0.1%)
Mental health^e^					
No	9,749 (87%)	9,689 (78%)	1,768 (65%)	2,781 (59%)	23,987 (78%)
Yes	1,516 (13%)	2,726 (22%)	983 (34%)	1,869 (40%)	7,094 (22%)
Missing	10 (<0.1%)	14 (<0.1%)	10 (0.4%)	11 (0.2%)	45 (0.1%)
Diabetes^d^					
No	10,551 (95%)	11,169 (91%)	2,347 (86%)	3,730 (81%)	27,797 (91%)
Yes	714 (5.1%)	1,251 (9.0%)	412 (14%)	926 (19%)	3,303 (9.2%)
Missing	10 (<0.1%)	9 (<0.1%)	2 (<0.1%)	5 (<0.1%)	26 (<0.1%)
Hypertension^d^					
No	8,495 (80%)	7,784 (67%)	1,457 (58%)	2,009 (46%)	19,745 (69%)
Yes	2,767 (20%)	4,628 (33%)	1,299 (41%)	2,646 (54%)	11,340 (31%)
Missing	13 (<0.1%)	17 (<0.1%)	5 (0.3%)	6 (<0.1%)	41 (0.1%)
Cigarette smoking status					
Current/former	983 (9.3%)	1,314 (12%)	424 (17%)	890 (21%)	3,611 (12%)
Never	10,292 (91%)	11,115 (88%)	2,337 (83%)	3,771 (79%)	27,515 (88%)
Missing	0 (0%)	0 (0%)	0 (0%)	0 (0%)	0 (0%)
Obesity					
Not obese	4,537 (39%)	4,202 (33%)	774 (29%)	1,239 (25%)	10,752 (34%)
Obese	6,738 (61%)	8,227 (67%)	1,987 (71%)	3,422 (75%)	20,374 (66%)
Missing	0 (0%)	0 (0%)	0 (0%)	0 (0%)	0 (0%)
Physical activity^f^					
Active	4,950 (46%)	6,144 (50%)	1,672 (60%)	3,166 (68%)	15,932 (52%)
Inactive	6,325 (54%)	6,285 (50%)	1,089 (40%)	1,495 (32%)	15,194 (48%)
Missing	0 (0%)	0 (0%)	0 (0%)	0 (0%)	0 (0%)

a All estimates are are weighted and account for the survey’s complex sampling design. Percentages may not sum to 100 due to rounding.

b Includes adults who received a General Educational Development high school equivalency diploma (GED).

c FPL is federal poverty level. Family income as a percentage of FPL is derived from the family’s income in the previous calendar year and family size using the U.S. Census Bureau’s poverty thresholds.

d Based on ever being told by a doctor or other health professional that the adult had the condition.

e Defined as having ever been diagnosed with depression or an anxiety disorder.

f Active is defined as meeting aerobic PA guidelines (≥150 min/week of moderate intensity, or ≥75 min/week of vigorous intensity, or an equivalent combination).

FPL, federal poverty level; PA, physical activity.

### 3.2 Association of neighborhood environment with the primary outcome (pain frequency)

As presented in Table 3, a higher Total Neighborhood Score (higher = more favorable) was significantly associated with lower cumulative odds of experiencing more frequent pain in the fully adjusted model (aOR = 0.95, 95% CI: 0.94–0.97). Each 1-point increase in the Total Neighborhood Score is associated with a 5% reduction in the cumulative odds of experiencing higher pain frequency, demonstrating its protective nature. To delineate the specific environmental drivers while accounting for conceptual overlap, the three functional domains were analyzed simultaneously in a Joint Domains Model. In this joint model, the Safety Domain demonstrated a robust, independent protective association against higher pain frequency (aOR = 0.84, 95% CI: 0.80–0.89). Leisure access also independently correlated with reduced pain frequency (aOR = 0.95, 95% CI: 0.91–1.00, *p* = 0.041), whereas the Access Domain did not reach statistical significance after mutual adjustment (Table 4). Sensitivity analyses utilizing the PPOM confirmed that the proportional odds assumption was not materially violated across severity thresholds (Supplementary Table S3).

**Table 3:** Survey-weighted ordinal logistic regression models evaluating the independent associations of neighborhood environment scores with pain frequency.

Exposure variable	Unadjusted model	Adjusted model
	OR (95% CI)	OR (95% CI)
Total Neighborhood Score (higher = more favorable)	0.89 (0.87–0.90)	0.95 (0.94–0.97)
Safety score (higher = safer)	0.74 (0.71–0.78)	0.84 (0.79–0.88)
Access score (higher = better access)	0.86 (0.84–0.88)	0.95 (0.93–0.98)
Leisure score (higher = more restorative)	0.78 (0.75–0.81)	0.92 (0.89–0.96)

OR, odds ratio; CI, confidence interval.

The unadjusted model includes only the respective exposure variable. The adjusted model controls for age, sex, educational attainment, family poverty level, region, urban/rural classification, marital status, arthritis, cancer, mental health diagnoses, diabetes, hypertension, smoking status, obesity, and physical activity. An OR < 1.00 indicates lower cumulative odds of experiencing more frequent pain.

**Table 4:** Joint domains model: multivariable survey-weighted ordinal logistic regression predicting pain frequency.

Domains analysis	Adjusted model	
Joint domains model	OR (95% CI)	*p*-value
Safety score (higher = safer)	0.84 (0.80–0.89)	<0.001
Access score (higher = better access)	0.97 (0.94–1.01)	0.143
Leisure score (higher = more restorative)	0.95 (0.91–1.00)	0.041

OR, odds ratio; CI, confidence interval.

In this joint model, the safety, access, and leisure scores were included simultaneously in a single equation to mutually adjust for conceptual overlap. The model is fully adjusted for age, sex, educational attainment, family poverty level, region, urban/rural classification, marital status, arthritis, cancer, mental health diagnoses, diabetes, hypertension, smoking status, obesity, and physical activity.

Moreover, the safety score, access score, and leisure score also evaluated, independently. Specifically, higher safety score had significantly lower odds of reporting more frequent pain (aOR = 0.84, 95% CI: 0.79–0.88). Higher access score was linked to decreased odds of experiencing more frequent pain (aOR = 0.95, 95% CI: 0.93–0.98). Likewise, higher leisure score was linked to decreased odds of experiencing more frequent pain (aOR = 0.92, 95% CI: 0.89–0.96).

Moreover, the full adjusted model results including all covariate estimates have provided in Supplementary Table S4.

### 3.3 Secondary pain outcomes (intensity, limitations, and family impact)

We further investigated the multidimensional impact of the neighborhood environment across secondary pain outcomes (Table 5). A higher Total Neighborhood Score (higher = more favorable) was consistently associated with decreased odds of pain limiting life/work activities (aOR = 0.97, 95% CI: 0.95–0.99) and impacting family life (aOR = 0.95, 95% CI: 0.93–0.97).

**Table 5:** Survey-weighted ordinal logistic regression models predicting secondary pain outcomes: pain intensity, activity limitations, and family impacts.

Exposure variable	Unadjusted model	Adjusted model
	OR (95% CI)	OR (95% CI)
Pain intensity		
Total Neighborhood Score (higher = more favorable)	0.99 (0.97–1.00)	1.00 (0.98–1.02)
Safety score (higher = safer)	0.90 (0.85–0.95)	0.96 (0.90–1.01)
Access score (higher = better access)	1.00 (0.97–1.03)	1.00 (0.97–1.04)
Leisure score (higher = more restorative)	0.98 (0.94–1.03)	1.01 (0.96–1.06)
Frequency of pain limits life/work		
Total Neighborhood Score (higher = more favorable)	0.97 (0.95–0.98)	0.97 (0.95–0.99)
Safety score (higher = safer)	0.80 (0.75–0.84)	0.89 (0.83–0.94)
Access score (higher = better access)	1.00 (0.98–1.03)	0.98 (0.95–1.01)
Leisure score (higher = more restorative)	0.91 (0.87–0.95)	0.93 (0.88–0.97)
Frequency of pain impacts family		
Total Neighborhood Score (higher = more favorable)	0.95 (0.93–0.97)	0.95 (0.93–0.97)
Safety score (higher = safer)	0.74 (0.70–0.79)	0.81 (0.76–0.86)
Access score (higher = better access)	0.98 (0.95–1.01)	0.97 (0.94–1.01)
Leisure score (higher = more restorative)	0.92 (0.87–0.97)	0.92 (0.87–0.98)

OR, odds ratio; CI, confidence interval.

Unadjusted models include only the single specific exposure variable. Adjusted models control for the full minimal sufficient adjustment set: age, sex, educational attainment, family poverty level, region, urban/rural classification, marital status, arthritis, cancer, mental health diagnoses, diabetes, hypertension, smoking status, obesity, and physical activity.

When evaluated in the Joint Domains Models, the safety score emerged as the most consistent independent protective factor across all secondary outcomes, significantly reducing the odds of severe pain intensity (aOR = 0.95), activity limitations (aOR = 0.89), and family impacts (aOR = 0.81). The leisure domain similarly demonstrated independent protective effects against activity limitations and family impacts, underscoring the comprehensive benefits of perceived neighborhood safety and recreational walkability on chronic pain burden (Supplementary Table S5).

Similarly, higher safety score had lower odds of reporting that pain limited their activities (aOR = 0.89, 95% CI: 0.83–0.94). Higher leisure score had lower odds of reporting that pain limited their activities (aOR = 0.93, 95% CI: 0.88–0.97). Higher safety score with those reporting access showing reduced odds of daily pain-related family impacts (aOR = 0.81, 95% CI: 0.76–0.86). Higher leisure score with those reporting access showing reduced odds of daily pain-related family impacts (aOR = 0.92, 95% CI: 0.87–0.98).

## 4 Discussion

This study utilized a nationally representative sample to examine the relationship between perceived neighborhood environment domains and the multidimensional burden of pain among U.S. adults. By employing a joint domains analytic strategy, we robustly accounted for the conceptual overlap among environmental features. Our findings demonstrate that individuals who perceived their neighborhoods as highly walkable and safe reported significantly lower pain frequency, fewer limitations in daily life, and reduced pain-related family impacts. Specifically, when analyzed jointly, perceived neighborhood safety and access to leisure spaces emerged as the strongest independent protective factors against chronic pain burden, persisting even after rigorous adjustment for a comprehensive set of sociodemographic, lifestyle, and clinical covariates.

The robust, independent protective effect of perceived safety (from crime and traffic) underscores the importance of the psychosocial dimensions of pain. Viewed through the lens of the biopsychosocial framework, living in environments perceived as unsafe fosters chronic psychological distress, fear-avoidance behaviors, and hypervigilance (15). This persistent state of arousal can contribute to allostatic load and central sensitization, directly amplifying pain severity and chronicity (16). Furthermore, fear of crime or traffic hazards can deter individuals from engaging in outdoor physical and social activities, initiating a vicious cycle of deconditioning, social isolation, and worsening musculoskeletal pain.

Interestingly, our joint domains model revealed divergent effects among different types of neighborhood amenities. While the Leisure domain (access to paths for relaxation and fun) remained independently protective against pain frequency and interference, the Access domain (proximity to shops and transit stops) did not retain statistical significance after mutual adjustment. This nuance highlights the complexity of the built environment. Access to commercial destinations and transit undoubtedly promotes utilitarian walking; however, these areas are often accompanied by “environmental burdens” such as traffic congestion, noise pollution, and crowding (17). Spaces dedicated to relaxation and leisure provide crucial opportunities for psychological restoration and stress buffering, which our data suggest may be more directly relevant to mitigating the subjective experience of pain.

These findings are consistent with our hypothesis that walkable and supportive environments may be linked to better physical wellbeing. Specifically, access to sidewalks and walking paths was associated with lower odds of experiencing pain, possibly reflecting the role of physical activity in pain management. Previous work supports this idea, suggesting that physical activity, often facilitated by walkable neighborhoods, can alleviate chronic pain conditions like osteoarthritis and fibromyalgia (18, 19). Additionally, access to places to relax, although broadly defined, was consistently associated with lower pain prevalence. These results support the notion that relaxing or restorative neighborhood spaces may contribute to pain relief, potentially by mitigating psychological stress, promoting movement, or providing calming sensory input. Studies show that green spaces and public parks can reduce stress and provide pain relief, particularly for individuals suffering from chronic pain (20, 21).

While the use of nationally representative data is a major strength, it is essential to acknowledge the inherent heterogeneity within such a vast sample. The relationships between perceived neighborhood features and pain may vary significantly across urban/rural divides, socioeconomic strata, and geographic regions. Although we rigorously adjusted for family poverty level, urbanicity, and region as covariates to isolate the main effects, aggregating data across highly variable environments might obscure critical subgroup-specific interactions. Future research should prioritize intersectional analyses to determine whether vulnerable populations, who often face both a higher baseline burden of pain and disproportionate exposure to adverse built environments, experience amplified health impacts (22).

This study has several important limitations that must be acknowledged. First, the cross-sectional design precludes any causal inference. Crucially, the issue of temporality and reverse causality cannot be ruled out: individuals experiencing frequent or high-impact pain may possess restricted mobility and heightened distress, leading them to appraise their neighborhood environment more negatively than their counterparts experiencing little to no pain interference counterparts (23). Second, we exclusively utilized the 2020 NHIS dataset because the detailed “Neighborhood Environment” module was uniquely administered in this cycle. However, the COVID-19 pandemic and associated lockdown mitigation measures significantly altered individuals’ interactions with their local environments, psychological wellbeing, and potentially overall survey response patterns during that year. Consequently, these pandemic-specific contexts should be considered when generalizing the findings. Third, all measures were self-reported. While subjective appraisal is theoretically crucial to the biopsychosocial model, relying solely on self-reports introduces shared method variance, which could inflate the observed correlations between neighborhood perceptions and pain outcomes. Integrating objective spatial data (e.g., GIS metrics) with subjective appraisals in future studies will yield a more holistic understanding of this dynamic. Fourth, the limitations and unique strengths of our pain measures warrant careful consideration. A notable strength of the 2020 NHIS pain module is its multidimensionality; by capturing frequency, severity, and functional interference, it aligns with the biopsychosocial framework better than unidimensional pain scales. Furthermore, these standardized items provide high external validity for national surveillance. However, as items measuring how often pain “limits” or “affects” daily activities capture functional restriction rather than the raw biological presence of pain, individuals with adaptive coping mechanisms or mild pain are necessarily captured in the “Never” reference categories. Consequently, our secondary models do not predict the crude presence of bodily nociception, which may underestimate the absolute prevalence of clinical pain. Finally, despite adjusting for a comprehensive minimal sufficient adjustment set (including specific mental health and chronic disease diagnoses), unmeasured confounding may still exist.

Despite these constraints, this study significantly advances the field by bridging spatial epidemiology and pain research. By moving beyond isolated univariable models and employing a rigorous joint domains approach, we minimized Type I error and clarified the specific environmental drivers of pain. Furthermore, our models comprehensively adjusted for objective clinical diagnoses (e.g., arthritis, cancer, mental health), preventing the over-adjustment bias commonly seen when using overly broad “general health” proxies.

## 5 Conclusion

In conclusion, our findings support the integration of the built environment into the biopsychosocial model of pain management. Perceived neighborhood safety and access to restorative leisure spaces are independently associated with reduced pain frequency and interference. These results suggest that clinical approaches to pain management should consider patients’ environmental contexts. Moreover, at the population level, interdisciplinary urban planning strategies aimed at traffic calming, crime reduction, and the expansion of accessible green spaces may serve as essential public health interventions to mitigate the widespread burden of pain.

## Data Availability

The original contributions presented in the study are included in the article/Supplementary material, further inquiries can be directed to the corresponding authors.

## Supplementary material

SUPPLEMENTARY FIGURE S1 Directed Acyclic Graph (DAG) demonstrating the conceptual framework and covariate selection for the multivariable models. Green nodes represent the exposures (neighborhood environment domains), the orange node represents the primary outcome (pain frequency), and purple nodes represent the predefined covariates. Arrows denote assumed causal pathways based on existing literature. This theoretical framework informed the minimal sufficient adjustment set used in all fully adjusted ordinal logistic regression models to effectively minimize overadjustment and confounding bias.
