# Supplementary material for: Perceived neighborhood environment and multidimensional pain burden among U.S. adults
Source: Front Public Health. 2026 Jul 8;14:1844301. doi: 10.3389/fpubh.2026.1844301 (PMC13388383; doi:10.3389/fpubh.2026.1844301)
Supplement: Supplementary file 1 [file Table_1.DOCX]

Supplementary Table S1. Frequencies and proportions of missing data for all variables included in the final analytical models (NHIS 2020)

| Variable | Missing number (percent) |
| --- | --- |
| Total Neighborhood Score | 1739 (5.5%) |
| Marital status | 978 (3.1%) |
| Frequency of pain | 442 (1.4%) |
| Educational attainment | 149 (0.5%) |
| Mental health | 55 (0.2%) |
| Hypertension | 51 (0.2%) |
| Arthritis | 43 (0.1%) |
| Cancer | 34 (0.1%) |
| Diabetes | 32 (0.1%) |
| Sex/gender | 2 (0.0%) |
| Age | 0 (0.0%) |
| Family income as a percentage of FPL | 0 (0.0%) |
| Urban-rural classification | 0 (0.0%) |
| Cigarette smoking status | 0 (0.0%) |
| Obesity | 0 (0.0%) |
| Physical activity | 0 (0.0%) |

NHIS, National Health Interview Survey; FPL, Federal Poverty Level.

Missing data were strictly addressed using Multiple Imputation by Chained Equations (MICE) prior to multivariable modeling.
