# Supplementary material for: Perceived neighborhood environment and multidimensional pain burden among U.S. adults
Source: Front Public Health. 2026 Jul 8;14:1844301. doi: 10.3389/fpubh.2026.1844301 (PMC13388383; doi:10.3389/fpubh.2026.1844301)
Supplement: Supplementary file 2 [file Table_2.DOCX]

Supplementary Table S2. Comparison of key variable distributions before and after Multiple Imputation by Chained Equations (MICE, m=20)

| Variable | Level/Category | Original Dataset (Before Imputation) | Pooled Imputed Datasets (After Imputation) |
| --- | --- | --- | --- |
|  | | Unweighted N (%) | Average Unweighted N (%) |
| **Core Exposure & Outcome** | | |  |
| Total Neighborhood Score | |  |  |
|  | Mean (SD) | 5.51 (2.02) | 5.52 (2.01) |
| Pain Frequency | |  |  |
|  | 0_Never | 11275 (36.2%) | 11444 (36.3%) |
|  | 1_Some | 12429 (39.9%) | 12604 (39.9%) |
|  | 2_Most | 2761 (8.9%) | 2800 (8.9%) |
|  | 3_Every | 4661 (15.0%) | 4720 (15.0%) |
| **Covariates (Highest Missing Rates)** | | |  |
| Family Poverty Level | |  |  |
|  | <100% FPL | 2824 (8.9%) | 2824 (8.9%) |
|  | >=200% FPL | 23523 (74.5%) | 23523 (74.5%) |
|  | 100-199% FPL | 5221 (16.5%) | 5221 (16.5%) |
| Obesity (BMI) | |  |  |
|  | Not Obese | 10945 (34.7%) | 10473 (33.2%) |
|  | Obese | 20623 (65.3%) | 21095 (66.8%) |
| Education Level | |  |  |
|  | <High School | 3125 (9.9%) | 3153 (10.0%) |
|  | Bachelor+ | 12365 (39.4%) | 12399 (39.3%) |
|  | High School | 6823 (21.7%) | 6864 (21.7%) |
|  | Some College | 9106 (29.0%) | 9152 (29.0%) |

SD, Standard Deviation; FPL, Federal Poverty Level; BMI, Body Mass Index.

The "Original Dataset" column reflects complete cases prior to imputation. The "Pooled Imputed Datasets" column reflects the averaged distributions across the 20 imputed datasets. Data were derived from the 2020 NHIS, conducted during the COVID-19 pandemic. Due to pandemic-related shifts to telephone interviewing and altered response rates, sampling weights were robustly adjusted by the National Center for Health Statistics (NCHS) to maintain national representativeness.
