# Supplementary material for: Perceived neighborhood environment and multidimensional pain burden among U.S. adults
Source: Front Public Health. 2026 Jul 8;14:1844301. doi: 10.3389/fpubh.2026.1844301 (PMC13388383; doi:10.3389/fpubh.2026.1844301)
Supplement: Supplementary file 3 [file Table_3.DOCX]

Supplementary Table S3. Sensitivity analysis: Partial proportional odds model (PPOM) evaluating the primary exposure (Total Neighborhood Score (higher = more favorable)) and pain frequency

| Threshold for Pain Frequency | Adjusted Odds Ratio | 95% CI |
| --- | --- | --- |
| Threshold 1: (Some/Most/Every) vs. (Never) | 0.97 | 0.95 - 0.99 |
| Threshold 2: (Most/Every) vs. (Never/Some) | 0.94 | 0.92 - 0.95 |
| Threshold 3: (Every) vs. (Never/Some/Most) | 0.91 | 0.89 - 0.93 |

CI, Confidence Interval; aOR, adjusted Odds Ratio.

The proportional odds assumption posits that the effect of the neighborhood score remains constant across all thresholds of pain frequency. This PPOM relaxes this strict constraint for the primary exposure. The aORs remain relatively consistent and in the same protective direction across all three severity thresholds, indicating that the standard ordinal logistic regression model used in the main analysis (Table 3) did not materially violate the proportional odds assumption. The model was fully adjusted for all sociodemographic and clinical covariates.
