# Supplementary material for: Perceived neighborhood environment and multidimensional pain burden among U.S. adults
Source: Front Public Health. 2026 Jul 8;14:1844301. doi: 10.3389/fpubh.2026.1844301 (PMC13388383; doi:10.3389/fpubh.2026.1844301)
Supplement: Supplementary file 4 [file Table_4.DOCX]

Supplementary Table S4. Complete multivariable survey-weighted ordinal logistic regression model predicting the primary outcome (pain frequency) with all covariate estimates (NHIS 2020).

| Variable group | Category level | Adjusted OR (95% CI) |
| --- | --- | --- |
| Primary Exposure | Per 1-point increase | 0.95 (0.94 - 0.97) |
| Age Group (Ref: 18-44) | 45-64 years | 1.39 (1.30 - 1.49) |
| Age Group (Ref: 18-44) | >=65 years | 1.21 (1.12 - 1.32) |
| Sex (Ref: Men) | Women | 0.98 (0.93 - 1.05) |
| Education Level (Ref: <High School) | Bachelor's degree or higher | 0.91 (0.81 - 1.02) |
| Education Level (Ref: <High School) | High school graduate | 0.94 (0.83 - 1.06) |
| Education Level (Ref: <High School) | Some college | 1.05 (0.93 - 1.18) |
| Family Poverty Level (Ref: <100% FPL) | >=200% FPL | 0.84 (0.74 - 0.95) |
| Family Poverty Level (Ref: <100% FPL) | 100-199% FPL | 0.85 (0.74 - 0.97) |
| US Region (Ref: Northeast) | Midwest | 1.26 (1.12 - 1.42) |
| US Region (Ref: Northeast) | South | 1.09 (0.97 - 1.22) |
| US Region (Ref: Northeast) | West | 1.17 (1.04 - 1.31) |
| Urbanicity (Ref: Rural) | Urban | 0.95 (0.86 - 1.04) |
| Marital Status (Ref: Married/Partnered) | Single/Other | 0.93 (0.87 - 0.99) |
| Arthritis History (Ref: No) | Yes (Arthritis) | 5.11 (4.77 - 5.48) |
| Cancer History (Ref: No) | Yes (Cancer) | 1.30 (1.19 - 1.42) |
| Mental Health Disorders (Ref: No) | Yes (Depression/Anxiety) | 2.24 (2.09 - 2.41) |
| Diabetes History (Ref: No) | Yes (Diabetes) | 1.38 (1.25 - 1.52) |
| Hypertension History (Ref: No) | Yes (Hypertension) | 1.42 (1.33 - 1.51) |
| Cigarette Smoking (Ref: Current/Former) | Never smoked | 0.62 (0.57 - 0.68) |
| Obesity status (Ref: Not Obese) | Obese | 1.16 (1.09 - 1.23) |
| Physical Activity Level (Ref: Active) | Inactive | 0.84 (0.79 - 0.89) |

NHIS, National Health Interview Survey; OR, Odds Ratio; CI, Confidence Interval; FPL, Federal Poverty Level; Ref, Reference Group.

Adjusted models control for the full minimal sufficient adjustment set: age, sex, educational attainment, family poverty level, region, urban/rural classification, marital status, arthritis, cancer, mental health diagnoses, diabetes, hypertension, smoking status, obesity, and physical activity.
