# Supplementary material for: Perceived neighborhood environment and multidimensional pain burden among U.S. adults
Source: Front Public Health. 2026 Jul 8;14:1844301. doi: 10.3389/fpubh.2026.1844301 (PMC13388383; doi:10.3389/fpubh.2026.1844301)
Supplement: Supplementary file 5 [file Table_5.DOCX]

Supplementary Table S5. Joint domains models: Multivariable survey-weighted ordinal logistic regression predicting secondary pain outcomes (intensity, limitations, and family impact)

| Domains analysis | Adjusted Model | |
| --- | --- | --- |
| Joint domains model | OR (95%CI) | P value |
| Pain intensity |  |  |
| Safety score (higher = safer) | 0.95 (0.90 - 1.01) | 0.093 |
| Access score (higher = better access) | 1.00 (0.97 - 1.04) | 0.864 |
| Leisure score (higher = more restorative) | 1.01 (0.96 - 1.06) | 0.725 |
|  |  |  |
| Frequency of pain limits life/work | |  |
| Safety score (higher = safer) | 0.89 (0.84 - 0.95) | <0.001 |
| Access score (higher = better access) | 1.01 (0.98 - 1.05) | 0.494 |
| Leisure score (higher = more restorative) | 0.92 (0.88 - 0.97) | 0.002 |
|  |  |  |
| Frequency of pain impacts family | |  |
| Safety score (higher = safer) | 0.81 (0.76 - 0.86) | <0.001 |
| Access score (higher = better access) | 1.00 (0.96 - 1.04) | 0.895 |
| Leisure score (higher = more restorative) | 0.94 (0.87 - 1.01) | 0.088 |

OR, Odds Ratio; CI, Confidence Interval.

For each secondary outcome, the safety, access, and leisure scores were entered simultaneously into a single multivariable equation to mutually adjust for conceptual environmental overlap. All models were fully adjusted for age, sex, educational attainment, family poverty level, region, urban/rural classification, marital status, arthritis, cancer, mental health diagnoses, diabetes, hypertension, smoking status, obesity, and physical activity.
