# Supplementary figures and images for: Perceived neighborhood environment and multidimensional pain burden among U.S. adults
Source: Front Public Health. 2026 Jul 8;14:1844301. doi: 10.3389/fpubh.2026.1844301 (PMC13388383; doi:10.3389/fpubh.2026.1844301)

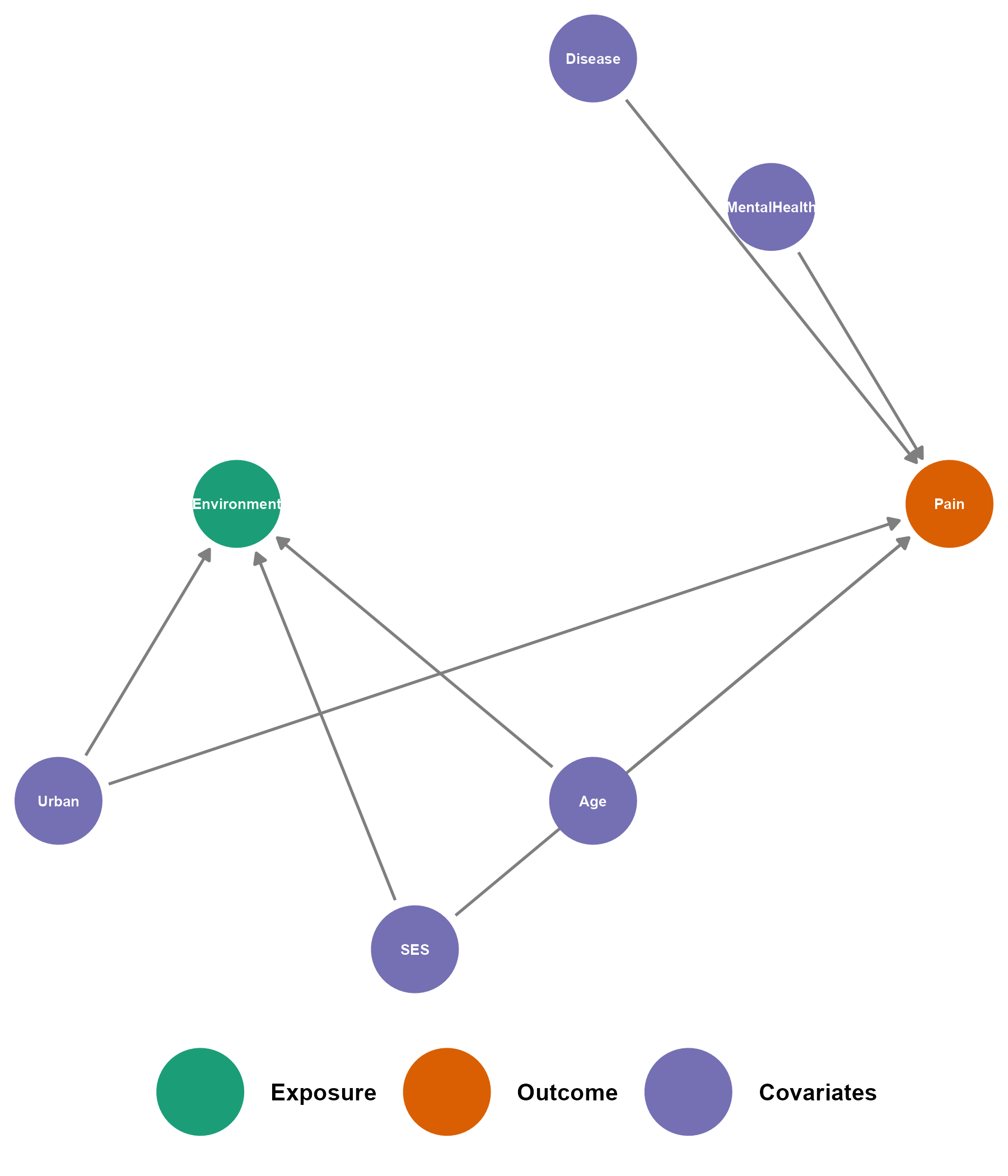

Supplement: SUPPLEMENTARY FIGURE S1 — Directed Acyclic Graph (DAG) demonstrating the conceptual framework and covariate selection for the multivariable models. Green nodes represent the exposures (neighborhood environment domains), the orange node represents the primary outcome (pain frequency), and purple nodes represent the predefined covariates. Arrows denote assumed causal pathways based on existing literature. This theoretical framework informed the minimal sufficient adjustment set used in all fully adjusted ordinal logistic regression models to effectively minimize overadjustment and confounding bias. [file Image_1.TIF]
